# Carbonic Anhydrase Inhibitors. Part 46^1^ Inhibition of Carbonic Anhydrase Isozymes I, II and IV With Trifluoromethylsulfonamide Derivatives and Their Zinc(II) and Copper(II) Complexes

**DOI:** 10.1155/MBD.1997.27

**Published:** 1997

**Authors:** Giovanna Mincione, Andrea Scozzafava, Claudiu T. Supuran

**Affiliations:** Università degli Studi Dipartimento di Chimica Laboratorio di Chimica Inorganica e Bioinorganica Via Gino Capponi 7 Florence I-50121 Italy

## Abstract

Reaction of aromatic/heterocyclic sulfonamides containing a free amino group with triflic anhydride afforded compounds possessing trifluoromethanesulfonamido moieties in their molecule. The Zn(II) and Cu(II) complexes of these new sulfonamides were prepared and characterized by standard procedures (elemental analysis, spectroscopic, magnetic, thermogravimetric and conductimetric measurements). The new derivatives showed good inhibitory activity against three isozymes of carbonic anhydrase (CA), i.e., CA I, II and IV.


### CARBONIC ANHYDRASE INHIBITORS. Part 46[1]
### INHIBITION OF CARBONIC ANHYDRASE ISOZYMES I, II AND IV WITH TRIFLUOROMETHYLSULFONAMIDE DERIVATIVES AND THEIR ZINC(II) AND COPPER(II) COMPLEXES

Giovanna Mincione, Andrea Scozzafava and Claudiu T. Supuran*

Università degli Studi, Dipartimento di Chimica, Laboratorio di Chimica Inorganica e Bioinorganica, Via Gino Capponi 7, I-50121, Florence, Italy

**Abstract**: Reaction of aromatic/heterocyclic sulfonamides containing a free amino group with triflic anhydride afforded compounds possessing trifluoromethanesulfonamido moieties in their molecule. The Zn(II) and Cu(II) complexes of these new sulfonamides were prepared and characterized by standard procedures (elemental analysis, spectroscopic, magnetic, thermogravimetric and conductimetric measurements). The new derivatives showed good inhibitory activity against three isozymes of carbonic anhydrase (CA), i.e., CA I, II and IV.

### Introduction

Sulfonamides possessing carbonic anhydrase (CA, EC 4.2.1.1) inhibitory properties such as acetazolamide **1** and some other thiadiazole-sulfonamides (benzolamide **2**, chlorzolamide **3**), methazolamide **4**, or trifluoromethazolamide **5**, together with compounds containing other ring systems[2] were used for the last 40 years in the treatment or prevention of glaucoma,[3,4] gastro-duodenal ulcers,[2] mountain sickness[5] and other conditions associated with acid-base disequilibria.[2,6]

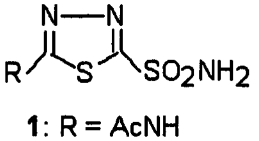

**1**: R = AcNH
**2**: R = $PhSO_2NH$
**3**: =

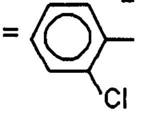

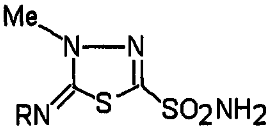

**4**: R = Ac
**5**: R = $CF_3CO$

$CF_3SO_2NH_2$

**6**

Recently, Maren and Conroy[7] discovered that polyhalogenated aliphatic sulfonamides such as trifluoromethanesulfonamide **6** and its congeners behave as extremely potent CA inhibitors against many of the eight CA isozymes presently isolated in higher vertebrates.[8] This was an extremely important discovery,[7] since previously it was universally accepted that only aromatic and heterocyclic sulfonamides possessing the general formula $RSO_2NH_2$ act as inhibitors of this enzyme,[2-4,9] with the aliphatic derivatives considered to be inactive.[3] Then, a large series of aliphatic sulfonamides with potent CA inhibitory properties was reported by a group from Merck, Sharp and Dohme.[10]

The X-ray crystal structure of the adduct of **6** with human CA II has also been reported,[11] this small sulfonamide binding directly to the zinc ion within the enzyme active site, as anion (Fig. 1), similarly to the aromatic and heterocyclic derivatives, for which X-ray crystallographic studies have been reported previously.[12-14] Practically the structures of the adducts of human CA II with acetazolamide **1**,[12] methazolamide **4**,[13] and the 4-amino-derivative of **2**, aminobenzolamide,[14] have been reported in the last years. These studies are of considerable interest for the design of more potent and selective (isozyme-specific) CA inhibitors.[2,6,11-14]

Fluoro-containing sulfonamides possessing CA inhibitory activity are of historical and practical importance, as the first compound for which topical antiglaucoma activity has been detected was trifluoromethazolamide **5**.[15] This compound could not be developed as a drug because of its chemical instability, as it hydrolyzes spontaneously in aqueous medium with a half life of 15 min. at the physiological pH.[15] However, trifluoromethazolamide remains an important lead molecule, as it demonstrated that sulfonamides possessing CA inhibitory activity may be administered topically as antiglaucoma agents, with a very good therapeutic effect, and without side effects.[4,15]

Recently, it was also reported[16-18] that metal complexes of heterocyclic sulfonamides such as **1-4** behave as even stronger CA inhibitors as compared to the ligands from which they derive. A large number of such complexes has been prepared, containing diverse main group and transition metal ions, and assayed as inhibitors of three CA isozymes CA I, II and IV, in the search for isozyme-specific inhibitors.

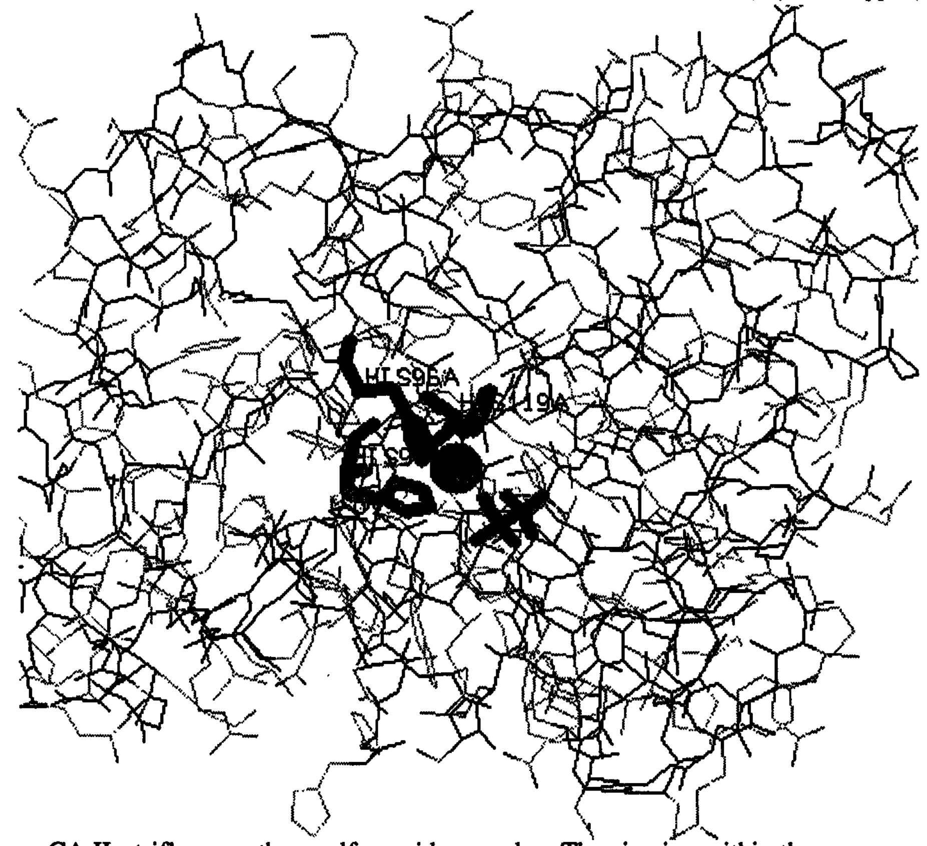

Fig. 1. Human CA II - trifluoromethanesulfonamide complex. The zinc ion within the enzyme active site, its three histidine ligands (His 94, 96 and 119) and the inhibitor molecule are evidenced. The figure was generated from the X-ray crystallographic coordinates of Hakansson and Liljas,[11] available from the Brookhaven Protein Database via Internet (file code 1bcd), by using the program RasWin 2.6.

Taking into account the interesting biological activity of fluoro-containing sulfonamides such as **5** and **6**, as well as the fact that inhibitors with the general formula: Aryl-$SO_2NH$-aryl'-$SO_2NH_2$ behave generally as very potent inhibitors,[1,19,20] we report in this paper fluoro-containing compounds of this type as well as their Zn(II) and Cu(II) complexes. The obtained derivatives were characterized by standard procedures and were assayed as inhibitors of isozymes CA I, II and IV, showing good activity.

### Materials and Methods

IR spectra were recorded on a Perkin-Elmer 16PC FTIR instrument, in the range 200-4000 $cm^{-1}$, in KBr pellets. Solution electronic spectra were recorded with a Cary 3 spectrophotometer interfaced with a PC. Electronic spectra were obtained by the diffuse reflectance technique in MgO as reference, with a Perkin Elmer Lambda 15 apparatus, in the range 200-900 $cm^{-1}$. Conductimetric measurements were done in DMF solutions, at 25°C (concentrations of 1 mM of complex) with a Fisher conductimeter. $^1H$-NMR spectra were recorded with a Bruker CPX-200 instrument working at 200 MHz, in DMSO-$d_6$ as solvent. Chemical shifts are expressed as $\delta$ values relative to $Me_4Si$ as external standard. EPR spectra were recorded on a Varian E-9 spectrometer at room temperature, in crystalline powder. The field was calibrated using crystalline diphenylpicrylhydrazyl (g = 2.0036). Magnetic susceptibility measurements were carried out at room temperature with a fully automated AZTEC DSM8 pendulum-type susceptometer. Mercury(II) tetrakis-(thiocyanato)cobaltate(II) was used as a susceptibility standard. Corrections for the diamagnetism were estimated from Pascal's constants.[21] Elemental analyses were done by combustion for C,H,N with an automated Carlo Erba analyzer, and gravimetrically for the metal ions, and were ± 0.4% of the theoretical values. Thermogravimetric measurements were done in air, at a heating rate of 10°C/min., with a Perkin Elmer 3600 thermobalance.

Acetazolamide and trifluoromethanesulfonamide used in the enzymatic assay as standards and in synthesis were from Sigma. Triflic anhydride **7** and solvents were from Merck or Acros. Sulfonamide **8e** was prepared as described in a previous paper by deacetylation of acetazolamide,[22] whereas other sulfonamides **8a-d** were commercially available from Aldrich, Sigma or Acros. Metal salts (zinc sulfate heptahydrate and copper(II) chloride dihydrate) were from Merck.

Human CA I and CA II cDNAs were expressed in *Escherichia coli* strain BL21 (DE3) from the plasmids pACA/HCA I and pACA/HCA II (the two plasmids were a gift from Prof. Sven Lindskog, Umea University, Sweden). Cell growth conditions were those described by Lindskog's group,[23] and enzymes were purified by affinity chromatography according to the method of Khalifah et al.[24] Enzyme concentrations were determined spectrophotometrically at 280 nm, using a molar absorptivity of 49 $mM^{-1}.cm^{-1}$ for CA I and 54 $mM^{-1}.cm^{-1}$ for CA II, respectively, based on $M_r$ = 28.85 kDa for CA I, and 29.3 kDa for CA II, respectively.[25] CA IV was isolated from bovine lung microsomes.[26]

Initial rates of 4-nitrophenyl acetate hydrolysis were monitored spectrophotometrically, at 400 nm and 25°C, with a Cary 3 apparatus interfaced with an IBM compatible PC by the method of Pocker and Stone.[27]. Solutions of substrate were prepared in anhydrous acetonitrile; the substrate concentrations varied between $10^{-2}$ and $10^{-6}$ M. A molar absorption coefficient $\varepsilon$ = 18,400 $M^{-1}.cm^{-1}$ was used for the 4-nitrophenolate formed by hydrolysis, in the conditions of the experiments (pH 7.80), as reported by Pocker and Stone.[27] Non-enzymatic hydrolysis rates were always subtracted from the observed rates. Duplicate experiments were done for each inhibitor, and the values reported throughout the paper are the averages of such results. $IC_{50}$ represents the molarity of inhibitor producing a 50% decrease of enzyme catalyzed hydrolysis of 4-nitrophenyl acetate.

**General procedure for the preparation of compounds 9**

An amount of 11.6 mmol of sulfonamide **8a-e** was suspended/dissolved in 50 mL of acetone. This mixture was magnetically stirred at 4°C for 30 min., then 1 mL (5.8 mmol) of triflic anhydride was added dropwise and the solution was stirred at 4°C overnight (when the same experimental procedure was utilized, but working at room temperature, the yield in trifluoromethylsulfonamides **9** was drastically reduced, and a large amount of resin was formed). Practically working at this molar ratio, the triflic acid **10** formed in the reaction is neutralized by the excess amino-sulfonamide. The solvent was evaporated then in vacuum and the brownish reaction mixture was taken up in 10 mL of cold water, when the triflates of amines **8a-e** being soluble, (in contrast to trifluoromethanesulfonamides **9a-e**) are easily separated from the desired products **9**. These were then recrystallized from acetone-water (9:1, v/v). Yields were in the range of 25-47 %. Of the five prepared compounds, four are new, whereas **9e** was previously reported by us, being synthesized by a variant of the above mentioned procedure.[28]

**General procedure for the preparation of complexes 11-20**

10 mmol of sulfonamide **9a-e** were suspended in 25 mL MeOH and the calculated amount of 1 N NaOH solution was added in order to obtain the monosodium salt. This was treated thereafter with an aqueous solution of the metal salt (Zn(II) sulfate and Cu(II) chloride, respectively), working at the molar ratios $M^{2+}$ : sulfonamide of 1:2. The reaction mixture was heated on a steam bath for 2 hours, then the precipitated complexes were filtered, thoroughly washed with cold water and alcohol. Crystallization was not done as the only solvents in which the complexes have good solubility are DMSO and DMF. The white powders (for the Zn(II) complexes), and the blue-greenish ones (in the case of the Cu(II) derivatives) **11-20** melt with decomposition at temperatures higher than 300 °C.

*4-(Trifluoromethylsulfonylamido)-benzenesulfonamide* **9a**, white crystals (yield: 47 %), m.p. 231-3 °C, IR (KBr), $cm^{-1}$: 710, 743, 786, 813, 1032, 1140 and 1178 ($SO_2^{sym}$), 1332 ($SO_2^{as}$), 3280 and 3400 (NH and $NH_2$); $^1$H-NMR (DMSO-$d_6$), $\delta$, ppm: 6.57 (br s, 2H, $NH_2$); $\nu_A$ = 6.90, $\nu_B$ = 7.18 (AA'BB' pattern, $J_{AB}$ 7.2 Hz, 4H, ArH from phenylene); 7.84 (br s, 1H, NH). Analysis, found: C: 27.4; H, 2.1; N, 9.2 %., $C_7H_7N_2F_3O_4S_2$ requires: C: 27.6; H, 2.3; N, 9.2 %.

*3-(Trifluoromethylsulfonylamido)-benzenesulfonamide* **9b**, white crystals (yield: 39 %), m.p. 188-91 °C, IR (KBr), $cm^{-1}$: 623, 738, 829, 957, 1040, 1096, 1145 and 1180 ($SO_2^{sym}$), 1316 ($SO_2^{as}$), 3280 and 3400 (NH and $NH_2$); $^1$H-NMR (DMSO-$d_6$), $\delta$, ppm: 6.50 (br s, 2H, $NH_2$); 7.10-7.51 (m, , 4H, ArH from 1,3-phenylene); 7.72 (m, 1H, NH). Analysis, found: C: 27.8; H, 2.0; N, 8.9 %., $C_7H_7N_2F_3O_4S_2$ requires: C: 27.6; H, 2.3; N, 9.2 %.

*4-(Trifluoromethylsulfonylamidomethyl)-benzenesulfonamide* **9c**, pale tan crystals (yield: 25 %), m.p. 197-8 °C, IR (KBr), $cm^{-1}$: 710, 748, 795, 816, 1042, 1154 and 1170 ($SO_2^{sym}$), 1324 ($SO_2^{as}$), 3280 and 3400 (NH and $NH_2$); $^1$H-NMR (DMSO-$d_6$), $\delta$, ppm: 4.88 (s, 2H, $SO_2NHCH_2$); 6.48 (br s, 2H, $NH_2$); 7.05 (m, AA'BB', $J_{AB}$ 7.3 Hz, 4H, ArH from phenylene); 7.84 (br s, 1H, NH). Analysis, found: C, 29.8; H, 2.9; N, 8.5 %; $C_8H_9N_2F_3O_4S_2$ requires: C, 30.1; H, 2.8; N, 8.8 %.

*4-(Trifluoromethylsulfonylamidoethyl)-benzenesulfonamide* **9d**, white crystals (yield: 28 %), m.p. 204-6 °C, IR (KBr), $cm^{-1}$: 688, 725, 840, 879, 937, 1035, 1090, 1155 and 1172 ($SO_2^{sym}$), 1325 ($SO_2^{as}$), 3280 and 3370 (NH and $NH_2$); $^1$H-NMR (DMSO-$d_6$), $\delta$, ppm: 3.10 (t, 2H, $\alpha CH_2$); 3.74 (t, 2H, $\beta CH_2$); 6.38 (br s, 2H, $NH_2$); $\nu_A$ = 6.90, $\nu_B$ = 7.21 (AA'BB' pattern, $J_{AB}$ = 7.3 Hz, 4H, ArH from phenylene); 7.69 (br s, 1H, NH). Analysis, found: C, 32.6; H, 3.0; N, 8.2 %; $C_9H_{11}N_2F_3O_4S_2$ requires: C, 32.5; H, 3.3; N, 8.4 %.

*5-(Trifluoromethylsulfonylamido)-1,3,4-thiadiazole-2-sulfonamide* **9e**, white crystals (yield: 32 %), m.p. 233-4 °C (dec.), lit.[28] m.p. 232 °C (dec.). IR (KBr), $cm^{-1}$: 490, 585, 643, 710, 907, 1030, 1130 and 1180 ($SO_2^{sym}$), 1330 ($SO_2^{as}$), 1425, 1590, 1620 (C=N), 3280 and 3390 (NH and $NH_2$); $^1$H-NMR (DMSO-$d_6$), $\delta$, ppm: 6.90 (br s, 2H, $NH_2$); 7.80 (br s, 1H, NH). Analysis, found: C, 11.2; H, 1.0; N, 17.8 %; $C_3H_3N_4F_3O_4S_3$ requires: C, 11.5; H, 0.9; N, 17.9 %.

**Results and Discussion**

Reaction of triflic anhydride **7** with amino-sulfonamides **8a-e** in cold acetone, in a molar ratio of 1:2 led to the trifluoromethylsulfonylamido-containing sulfonamides **9a-e** and triflic acid **10**, which was neutralized by the excess amino-sulfonamide used in the synthesis (Scheme 1).

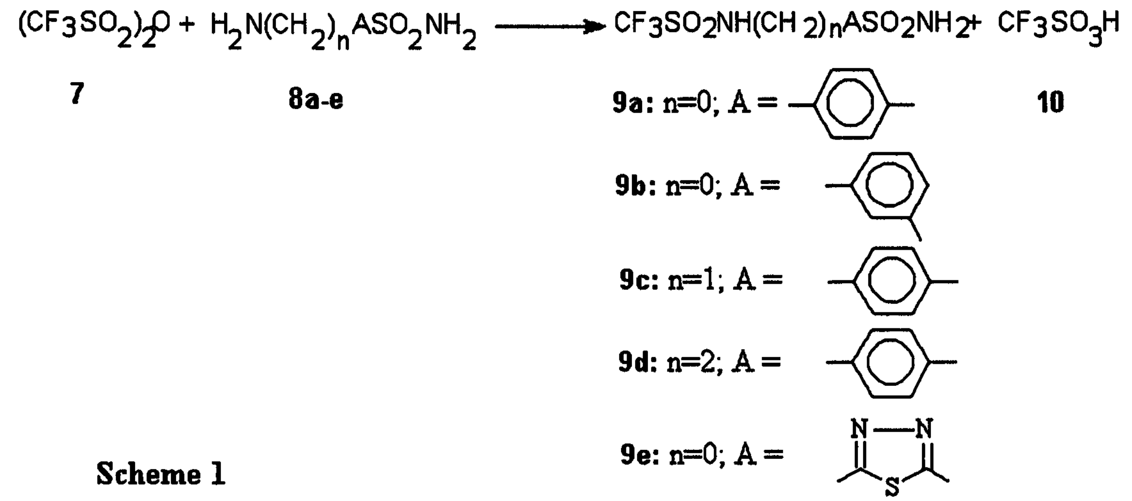

**Scheme 1**

The new compounds **9** have been characterized by elemental analysis (± 0.4 % of the theoretical values, calculated for the proposed formulas) and spectroscopic methods (IR, UV and $^1$H-NMR spectroscopy - see Materials and Methods for details) that confirmed the proposed structures.

The Zn(II) and Cu(II) complexes **11-20** containing the conjugate bases of sulfonamides **9a-e** as ligands, prepared in the present study, and their elemental analysis data are presented in Table I (**La-Le** stand for the sulfonamide ($SO_2NH$ moiety) deprotonated species of compounds **9a-9e**, respectively).

Some spectroscopic (IR and $^1$H-NMR), magnetic and conductimetric data for the newly synthesized complexes are shown in Table II.

In the IR spectra of the complexes **11-20**, the following features were evidenced: (i) the shift of the sulfonamido vibrations (the first symmetrical one, attributed[19,28] to the $SO_2NH$ moiety, as well as the antisymmetrical vibration) with 10-28 $cm^{-1}$ towards lower wavenumbers as compared to the corresponding vibrations from the IR spectra of sulfonamides **9a-e**, proving the involvement of these moieties in the interaction with the metal ions;[16-18] (ii) the lack of the ν(NH) vibrations from 3280 $cm^{-1}$ in the IR spectra of sulfonamides **9a-e**, whereas the vibrations around 3400 $cm^{-1}$ are present both in the spectra of the original sulfonamides as well as those of the metal complexes (data not shown); (iii) the appearance of broad ν(OH) bands due to the presence of coordinated water molecules, over 3400 $cm^{-1}$ (the only exception is constituted by the derivative **15**, which does not contain water); (iv) for the thiadiazole derivatives **15** and **20**, the C=N vibrations, appearing at 1620 $cm^{-1}$ in the spectrum of **9e**, are shifted to 1600 $cm^{-1}$ in the spectra of the complexes; (v) the presence of ν(M-N) and/or ν(M-O) bands in the region 200-400 $cm^{-1}$ of the spectra, for complexes **11-20**, vibrations not present in the spectra of the ligands (data not shown).

In the $^1$H-NMR spectra of complexes **11-20**, the signals of the $SO_2NH$ proton is absent, whereas those of the $SO_2NH_2$ protons (for the diamagnetic Zn(II) derivatives **11-15**) are slightly shifted with 0.02-0.09 ppm towards lower field as compared to the corresponding signals of the original sulfonamides, whereas the signals of the other protons remain unchanged. For the Cu(II) derivatives **16-20**, all the signals in the $^1$H-NMR spectra are isotropically shifted over 12 ppm, due to the effect of the paramagnetic ions.

Solution electronic spectra of the complexes were quite similar to the corresponding spectra of the monosodium salts of sulfonamides **9a-e** (data not shown), proving the presence of the deprotonated sulfonamido moieties in their molecule. As the $SO_2NH$ group is more acidic than the $SO_2NH_2$ one in derivatives **9a-e**,[19] it is obvious that in the presence of one equivalent of base it is the first one to be deprotonated, and as seen from the IR and $^1$H-NMR data presented above, this is the moiety interacting primarily with the metal ions in the prepared complexes.

Magnetic moments of the Cu(II) complexes were in the range of 1.88-1.96 BM, which correlated with the presence of a large, structureless band in the range 16,500 - 16,850 $cm^{-1}$ in the reflectance diffuse spectra and axial EPR spectra with the parameters $g_\perp$ = 2.06-2.07, and $g_\parallel$ = 2.35-2.37 (data not shown), suggest an octahedral surrounding of Cu(II) in complexes **16-20**.[29]

Table I: Prepared complexes **11-20**, containing the conjugate base of sulfonamides **9** and their elemental analysis data (**La-Le** stand for the sulfonamide ($SO_2NH$ moiety) deprotonated species of compounds **9a-9e**, respectively).

| No. | Complex | Yield (%) | %M[a] | Analysis (calculated/found) %C[b] | %H[b] | %N[b] | $\%H_2O$[c] |
|---|---|---|---|---|---|---|---|
| **11** | $[Zn(La)_2(OH_2)_2]$ | 63 | 9.2/9.5 | 23.7/23.5 | 2.2/1.8 | 7.9/7.5 | 5.0/5.1 |
| **12** | $[Zn(Lb)_2(OH_2)_2]$ | 72 | 9.2/9.3 | 23.7/23.6 | 2.2/2.3 | 7.9/7.8 | 5.0/4.8 |
| **13** | $[Zn(Lc)_2(OH_2)_2]$ | 83 | 8.9/8.8 | 26.1/25.7 | 2.7/2.9 | 7.6/7.3 | 4.9/5.0 |
| **14** | $[Zn(Ld)_2(OH_2)_2]$ | 79 | 8.5/8.1 | 28.3/28.1 | 3.1/3.3 | 7.3/6.9 | 4.7/4.5 |
| **15** | $[Zn(Le)_2]$ | 94 | 9.5/9.4 | 10.4/10.5 | 1.3/1.3 | 16.2/16.0 | d |
| **16** | $[Cu(La)_2(OH_2)_4]$ | 69 | 8.5/8.7 | 22.6/22.3 | 2.7/2.7 | 7.5/7.1 | 9.7/9.7 |
| **17** | $[Cu(Lb)_2(OH_2)_4]$ | 85 | 8.5/8.8 | 22.6/22.6 | 2.7/2.6 | 7.5/7.4 | 9.7/9.5 |
| **18** | $[Cu(Lc)_2(OH_2)_4]$ | 73 | 8.2/7.9 | 24.9/25.2 | 3.1/2.9 | 7.2/7.0 | 9.3/9.2 |
| **19** | $[Cu(Ld)_2(OH_2)_4]$ | 95 | 7.9/7.7 | 27.0/26.7 | 3.5/3.4 | 7.0/6.9 | 9.0/9.1 |
| **20** | $[Cu(Le)_2(OH_2)_2]$ | 62 | 8.8/8.7 | 9.9/9.8 | 1.1/1.1 | 15.5/15.1 | d |

[a]By gravimetry; [b]By combustion; [c]By TG analysis, lost in one step at temperatures in the range 170-180 °C; [d]No weight loss under 250 °C evidenced.

Table II: IR, $^1$H-NMR, and reflectance diffuse spectral data, as well as magnetic moments at room temperature, for complexes **11-20**.

| **Cpd.** | **IR. Spectra** [a], ($cm^{-1}$) $\Delta(SO_2)^s$ | $\Delta(SO_2)^{as}$ | **$^1$H-NMR Spectra**[b] $SO_2NH_2$, δ (ppm) | $\mu_{eff}$[c], (BM) | **RD Spectra** [d] ($cm^{-1}$) |
|---|---|---|---|---|---|
| **11** | 13 | 15 | 6.59 | e | g |
| **12** | 10 | 15 | 6.55 | e | g |
| **13** | 11 | 18 | 6.54 | e | g |
| **14** | 21 | 20 | 6.47 | e | g |
| **15** | 20 | 17 | 6.96 | e | g |
| **16** | 14 | 24 | f | 1.95 | 16,820 |
| **17** | 12 | 19 | f | 1.95 | 16,770 |
| **18** | 17 | 21 | f | 1.93 | 16,500 |
| **19** | 23 | 27 | f | 1.96 | 16,800 |
| **20** | 22 | 28 | f | 1.88 | 16,850 |

[a] In KBr pellets; $\Delta(SO_2)_f = (SO_2)_{sulfonamide} - (SO_2)_{complex}$; [b] In DMSO-$d_6$; [c] At room temperature; [d] In MgO as reference [e] Diamagnetic; [f] Isotropically shifted signal due to the presence of the paramagnetic ion at chemical shifts over 12 ppm; [g]No transitions evidenced.

All the prepared complexes **11-20** had a non-electrolyte behavior in DMF and DMSO solutions, with molar conductivities at 25 °C in the range of 2.5 - 5.0 $^{-1}$ . $cm^2$ . $mol^{-1}$ (data not shown).

From the above data it can be concluded that compounds **9a-d** probably act as monodentate ligands when deprotonated, by means of the secondary sulfonamido moiety ($SO_2NH$), whereas in the case of the thiadiazole-sulfonamide **9e**, in addition to the above mentioned group, some endocyclic heteroatoms (such as N-2 or N-3) probably participate to coordination. The metal ions possess a tetrahedral geometry in the Zn(II) derivatives **11-15**, and an octahedral one for the Cu(II) derivatives **16-20**, with water molecules occupying the remaining coordination positions. Proposed structures for the prepared complexes are shown below. Some ambiguity remains regarding the structure of the thiadiazole-sulfonamide containing complexes **15** and **20**.

Table III: Biological activity data of sulfonamide CA inhibitors and their metal complexes ($IC_{50}$ - the mean of two different assays - represents the molarity of inhibitor producing a 50% decrease of enzyme specific activity for the *p*-nitrophenyl acetate hydrolysis reaction)[27].

Previous work on related ligands, such as acetazolamide **1**,[16,17a,c] benzolamide **2**,[18d,30] or chlorzolamide **3**,[18d] proved the coordination versatility of these compounds, with practically all their heteroatoms being able to interact with the metal ions. Still, some donor systems are preferred: in most situations, compounds **1-3** interact with metal ions in deprotonated state, bidentately, by means of the sulfonamido nitrogen and the endocyclic N-2 atom.[16,17,30] Of the three ligands mentioned above, **9e** is more similar to benzolamide **2**, which has the most complicated coordination chemistry: the X-ray crystal structures for some of its metal complexes were recently obtained,[30] proving that benzolamide is doubly deprotonated at both sulfonamido moieties, and interacts with the metal ions by means of the two sulfonamido nitrogens as well as an endocyclic nitrogen atom (N-3). Thus, we predict for **9e** a bidentate or bridging bidentate ligand behaviour, as depicted schematically below.

| Compound | IC$_{50}$ (μM) CA I[a] | CA II[a] | CA IV[b] |
|---|---|---|---|
| **1 (acetazolamide)** | 90.0 | 1.1 | 22.5 |
| **6 ($CF_3SO_2NH_2$)** | 15.8 | 0.5 | 6.9 |
| **9a** | 165.5 | 10.9 | 64.6 |
| **9b** | 188.4 | 18.7 | 95.3 |
| **9c** | 137.8 | 5.0 | 24.5 |
| **9d** | 81.3 | 0.6 | 18.4 |
| **9e** | 59.8 | 0.1 | 3.2 |
| **11** | 120.1 | 8.0 | 41.7 |
| **12** | 150.9 | 9.5 | 44.3 |
| **13** | 94.1 | 2.8 | 17.7 |
| **14** | 43.5 | 0.3 | 12.8 |
| **15** | 39.3 | 0.01 | 1.5 |
| **16** | 97.2 | 6.3 | 29.8 |
| **17** | 133.6 | 6.1 | 63.9 |
| **18** | 66.2 | 2.0 | 10.6 |
| **19** | 37.5 | 0.2 | 9.1 |
| **20** | 32.6 | 0.01 | 1.1 |

[a]Human (cloned) isozyme; [b]Isolated from bovine lung microsomes.[26]

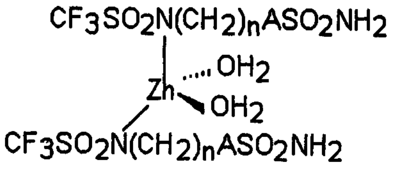

**11-14**

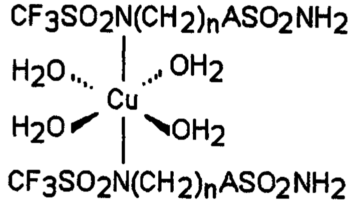

**16-19**

Unfortunately no good crystals of derivatives **15** and **20** were available for X-ray crystallography, so that the structures proposed for these two complexes are tentative.

CA inhibition data with compounds **9a-e**, **11-20** as well as standard inhibitors (acetazolamide **1** and trifluoromethanesulfonamide **6**), against three isozymes, CA I, II and IV, are shown in Table III.

The three investigated isozymes have very different susceptibilities to be inhibited by sulfonamides, with CA II being the most sensible, followed by CA IV, whereas CA I possesses the lowest affinity for this class of inhibitors.[2-4] This trend is also observed for the compounds reported in this study. More than that, the aromatic derivatives **9a-d** (and their metal complexes) were less active than the heterocyclic derivative **9e** (and its metal complexes). The most inactive inhibitor was the 3-amino-benzenesulfonamide derivative **9b**, whereas *para*-substituted compounds had better inhibitory properties. For these last derivatives, inhibitory efficiency increased with *n*, from the sulfanilamide derivative **9a** to the aminoethyl one (n=2) **9d**, a trend which was also conserved for the corresponding metal complexes, for all three CA isozymes. The copper complexes were more active than the corresponding zinc derivatives, which in turn were more inhibitory than the sulfonamides from which they were prepared. This can be correlated with the inhibitory effect of the metal ions contained in these compounds, as it was shown by us that they bind to the histidine cluster at the entrance of the CA II active site,[31] or to histidine residues situated in a solvent-exposed region for the other isozymes.[32] Although the sulfonamides **9a-c** are weaker inhibitors than acetazolamide, already derivatives **9d** and **9e** as well as some of their metal complexes (**14**, **15**, **19**, **20**) show strong affinities for all the investigated CA isozymes.

As mentioned in the introductory section, a major drawback of the previously reported[15] fluoro-containing sulfonamides was their chemical instability. Thus, trifluoromethazolamide **5** spontaneously hydrolyzes in aqueous medium to 4-methyl-5-imino-2-sulfonamido-$\delta^2$-1,3,4-thiadiazoline and trifluoro-acetate, with a half-life of 15 min.[15] Thus, we have tested the stability of compounds reported in this study, containing the trifluoromethylsulfonamido moieties in their molecule, in order to determine whether hydrolysis of the type reported for trifluoromethazolamide **5** occurs. By means of thin layer chromatography, no hydrolysis products have been detected in aqueous solutions of derivatives **9a-e** (in concentration ranges of 0.1 μM - 1 mM) after periods as long as 1 week (the solutions were monitored initially each 4 hours, and after the first day, each 24 hours).

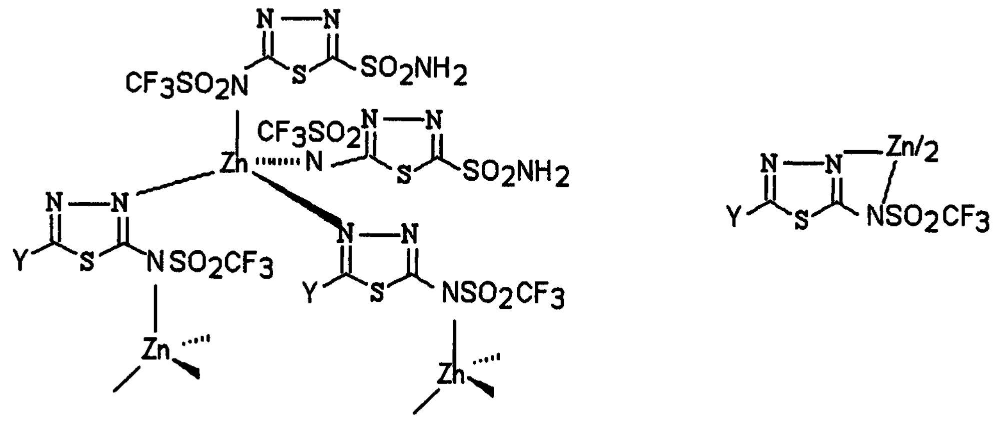

**15**: Y = $SO_2NH_2$

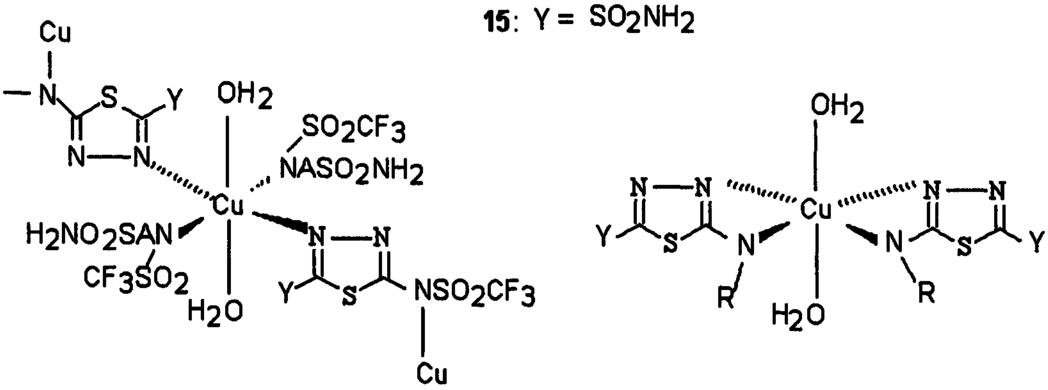

**20**: Y = $SO_2NH_2$; R = $CF_3SO_2$; A = 

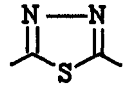

More than that, the CA inhibitory potency of these solutions remained constant in time, proving again that hydrolysis does not occur. Thus, the compounds reported by us here have an important advantage over trifluoromethazolamide: in addition of being very strong CA inhibitors, they are chemically stable compounds.

**Acknowledgments**: This research was financed in part by the EU grant ERB CIPDCT 940051. We are indebted to Dr. S. Lindskog (Umea University, Sweden) for the gift of the plasmids expressing CA I and CAII and to Drs. T.H. Maren and C.W. Conroy (University of Florida, Gainesville) for stimulating discussions.

**References**

1. Preceding part: C.T. Supuran, F.Briganti, A.Scozzafava, *J. Enzyme Inhibition.*, in press.
2. a) C.T. Supuran, *Roum.Chem.Quart.Rev.*, **1993**, *1*, 77-116; b) C.T. Supuran, "Carbonic anhydrase inhibitors", in "Carbonic Anhydrase and Modulation of Physiologic and Pathologic Processes in the Organism", I. Puscas Ed., Helicon, Timisoara **1994**, pp. 29-111; c) I. Puscas, C.T. Supuran, "Farmacologia clinica da ulcera peptica" in "Aparelho Digestivo", J. Coelho Ed., MEDSI, Rio de Janeiro, **1996**, pp. 1704-1734.
3. T.H. Maren, *Pharmacol.Rev.*, **1967**, *47*, 595-782.
4. T.H. Maren, *J. Glaucoma*, **1995**, *4*, 49-62.
5. E.B. Larson, R.C. Roach, R.B. Schoene, T.F. Hornbein, *JAMA*, **1982**, *248*, 328-332.
6. S. Lindskog, P.J. Wistrand. " Inhibition of carbonic anhydrase ", in "Design of Enzyme Inhibitors as Drugs", M.J. Sandler, H.J. Smith Eds., Oxford Univ. Press, Oxford, **1987**, pp. 698-723.
7. T.H. Maren, C.W. Conroy, *J.Biol.Chem.*, **1993**, 268, 26233-26238.
8. D. Hewett-Emmett, R.E. Tashian, *Mol.Phylogenet.Evol.*, **1996**, *5*, 50-77.
9. T.H. Maren, *Annu.Rev.Pharmacol.Toxicol.*, **1976**, *16*, 309-327.
10. T.H.Scholz, J.M. Sondey, W.C. Randall, H. Schwam, W.J. Thompson, P.J. Mallorga, M.F. Sugrue, S.L. Graham, *J.Med.Chem.*, **1993**, *36*, 2134-2141.

11. K. Hakansson, A. Liljas, *FEBS Lett.*, **1994**, *350*, 319-322.
12. J. Vidgren, A. Liljas, N.P.C. Walker, *Int.J.Biol.Macromol.*, **1990**, *12*, 342-344.
13. S. Chakravarty, K.K. Kannan, *J.Mol.Biol.*, **1994**, *243*, 298-309.
14. J. Vidgren, A. Svensson, A. Liljas *Int. J. Biol. Macromol.*, **1993**, *15*, 97-100
15 T.H. Maren, L. Jankowska, G.F. Edelhauser, G. Sanyal, *Exp.Eye Res.*, **1983**, *36*, 457-480.
16. a) G. Alzuet, S. Ferrer, J. Borras, C.T. Supuran, *Roum.Chem.Quart.Rev.*, **1994**, *2*, 283-300; b) C.T. Supuran, R. Stefan, G. Manole, I. Puscas, M. Andruh, *Rev.Roum.Chim.*, **1991**, *36*,1175-1179; c) C.T. Supuran, G. Manole, M. Andruh, *J.Inorg.Biochem.*, **1993**, *49*, 97-103; d) C.T. Supuran, *Rev.Roum.Chim.*, **1992**, *37*, 849-855; e) C.T. Supuran, M. Andruh, *Rev.Roum.Chim.*, **1994**, *39*, 1229-1234.
17. a) C.T. Supuran, *Rev.Roum.Chim.*, **1993**, *38*, 229-236; b) S.L. Sumalan, J. Casanova, G. Alzuet, J. Borras, A. Castiñeiras, C.T. Supuran, *J.Inorg.Biochem.*, **1996**, 62, 31-39; c) C.T. Supuran, G.L. Almajan, *Main Group Met. Chem.*, **1995**, *18*, 347-351.
18. a) C.T. Supuran, *Metal Based Drugs*, **1995**, 2, 327-330; b) C.T. Supuran, *Metal Based Drugs*, **1995**, *2*, 331-336; c) J. Borras, T. Cristea, C.T. Supuran, *Main Group Met. Chem.*, **1996**, *19*, 339-346; d) J. Borras, J. Casanova, T. Cristea, A. Gheorghe, A. Scozzafava, C.T. Supuran, V. Tudor, *Metal Based Drugs*, **1996**, *3*, 143-148.
19. a) C.T. Supuran, M.A. Ilies, T.B. Tewson, E.R. Swenson, *J.Med.Chem.*, in press; b) A. Scozzafava, C.T. Supuran, *J.Enzyme Inhibition*, in press.
20. a) J.R. Vaughan, J.A. Eichler, G.W. Anderson, *J.Org.Chem.*, **1956**, *21*, 700-701; b) R.W. Young, K.H. Wood, J.R. Vaughan, G.W. Anderson, *J.Am.Chem.Soc.*, **1956**, *78*, 4649-4654.
21. R.S. Drago, in "Physical Methods in Chemistry", W.B. Saunders & Co., London, **1977**, p. 411.
22. A. Jitianu, M.A. Ilies, A. Scozzafava, C.T. Supuran, *Main Group Met. Chem.*, **1997**, 20, 147-153.
23.a) C. Forsman, G. Behravan, A. Osterman, B.H. Jonsson, *Acta Chem. Scand.*, **1988**, *B42*, 314-318; b) G. Behravan, P. Jonasson, B.H. Jonsson, S. Lindskog, *Eur.J.Biochem.*, **1991**, *198*, 589-592.
24. R.G. Khalifah, D.J. Strader, S.H. Bryant, S.M. Gibson, *Biochemistry*, **1977**, *16*, 2241-2247.
25. a) P.O. Nyman, S. Lindskog, *Biochim.Biophys.Acta*, **1964**, *85*, 141-151; b) L.E. Henderson, D. Henriksson, P.O. Nyman, *J.Biol.Chem.*, **1976**, *251*, 5457-5463.
26. T.H. Maren, G.C. Wynns, P.J. Wistrand, *Mol. Pharmacol.*, **1993**, *44*, 901-906
27. Y. Pocker, J.T. Stone, *Biochemistry*, **1967**, *6*, 668-679.
28. C.T. Supuran, M.D. Banciu, A. Popescu, *Rev.Roum.Chim.*, **1992**, *37*, 289-297.
29. B.J. Hathaway, "Copper", in "Comprehensive Coordination Chemistry", Vol. 5, G. Wilkinson, R.D. Gillard, J. Cleverty Eds., Pergamon, New York, 1987, pp. 533-546.
30. J.Borras, G.Alzuet, J.Casanova, C.T.Supuran, manuscript in preparation.
31. C.T. Supuran, *Main Group Met. Chem.*, **1996**, *19*, 347-354.
32. F. Briganti, S. Mangani, P. Orioli, A. Scozzafava, C.T. Supuran, *Biochemistry*, in press.

**Received: January 17, 1997 - Accepted: January 30, 1997 - Received in revised camera-ready format: January 31, 1997**

